# Indoor Air Quality at Home—An Economic Analysis

**DOI:** 10.3390/ijerph18041679

**Published:** 2021-02-09

**Authors:** Amy Dymond, Stuart Mealing, Jessica McMaster, Hayden Holmes, Lesley Owen

**Affiliations:** 1 York Health Economics Consortium, Enterprise House, Innovation Way, University of York, Heslington, York YO10 5NQ, UK; stuart.mealing@york.ac.uk (S.M.); jm2309@cam.ac.uk (J.M.); hayden.holmes@york.ac.uk (H.H.); 2 National Institute for Health and Care Excellence, 10 Spring Gardens, London SW1A 2BU, UK; lesley.owen@nice.org.uk

**Keywords:** indoor air pollution, public health guidance, economic model, cost-effectiveness analysis

## Abstract

Background: People with respiratory conditions are susceptible to health problems caused by exposure to indoor air pollutants. An economic framework was developed to inform a guideline developed by National Institute for Health and Care Excellence (NICE) to estimate the required level of efficacy necessary for an intervention to be cost-saving in dwellings across England. Methods: An economic modelling framework was built to estimate the incremental costs pre- and post-implementation of interventions designed to reduce exposure to indoor air pollution within dwellings of varying building-related risk factors and profiles. The intervention cost was varied simultaneously with the relative reduction in symptomatic cases of each health condition to estimate the point at which an intervention may become cost-saving. Four health conditions were considered. Results: People living in dwellings with either an extreme risk profile or usable floor area <90 m^2^ have the greatest capacity to benefit and save National Health Service (NHS) costs from interventions at any given level of effectiveness and upfront cost. Conclusions: At any effectiveness level, the threshold for the upfront intervention cost to remain cost-saving is equivalent across the different home characteristics. The flexible model can be used to guide decision-making under a range of scenarios.

## 1. Introduction

People in Europe spend up to 90% of their lives indoors and 66% of this time is spent at home [1]. Indoor air quality has an impact on multiple health outcomes, including respiratory and cardiovascular illness, allergic symptoms, cancers and premature mortality [2,3]. Common pollutants include particulate matter (PM_2.5_ and PM_10_); nitrogen dioxide (NO_2_); and volatile organic compounds (VOCs) including benzene, formaldehyde and polycyclic aromatic hydrocarbons (PAHs). Sources of these pollutants include smoking, cooking, heating, unvented gas heaters and cookers, solvent use, renovations, new furniture, household products and biological sources (e.g., moulds) [1,3,4].

Common building problems such as damp are a known risk factor for health conditions. For example, a systematic review of 31 studies showed that exposure to damp was related to increased risk of all types of rhinitis [4]. High indoor particulate matter, NO_2_ and VOC levels were typically associated with respiratory symptoms, particularly asthma symptoms in children [3]. A systematic review of studies from 123 countries showed that the relative risk (RR) for asthma associated with the use of polluting fuels and technologies was 1.23 (95% CI: 1.11–1.36), and the RR for chronic obstructive pulmonary disease (COPD) was 1.70 (95% CI: 1.47–1.97) [2]. Therefore, children and people with either respiratory, cardiovascular, or both conditions are considered most vulnerable to health problems caused by indoor air quality [1].

While it is known that exposure to indoor pollutants affects health, there is little data to demonstrate the impact that alleviating these sources of pollution would have on respiratory and cardiovascular diseases. A global study has shown that between 2000 and 2017, deaths and disease burden associated with household air pollution steadily reduced, by 36% (95% CI 29–43) and 30% (95% CI 25–36), respectively. The greatest relative reduction in disability-adjusted life years (DALYs) occurred in the European region (71%). The reason for this reduction in morbidity is postulated to be cleaner fuels and better cooking technology, although the evidence for this is not consistent [2].

Nurmagambetow et al. (2011) systematically reviewed economic evaluations on the efficiency of home-based multicomponent interventions, with a focus to improve asthma-related morbidity outcomes. Interventions included home visits by trained personnel to assess and reduce adverse effects of indoor environmental pollutants, and educating households on how to reduce exposure to asthma triggers. The results implied that for every dollar spent on the intervention, the monetary value of the resulting benefits, such as averted medical costs or averted productivity losses, was $5.30–$14.00 (in 2007 US$) [5].

Interventions to reduce indoor air pollution need not be expensive. For example, ventilation is a predictor of indoor NO_2_, and PAH levels are higher in smoking households [3]. Therefore, free interventions such as smoking cessation and opening windows could reduce the levels of these pollutants.

In the UK, a National Institute for Health and Care Excellence (NICE) Public Health Advisory Committee (PHAC) developed guidelines to reduce exposure to pollutant sources and emissions within dwellings [6]. The NICE guidelines were aimed at a varied audience including environmental health practitioners, architects, public health professionals, planners/regulators involved with residential developments, private landlords and housing associations. The development of public health guidelines involves systematic identification and consideration of effectiveness and cost-effectiveness evidence, to facilitate the recommendation of interventions which are demonstrated to be effective and value for money [7]. The review of effectiveness evidence conducted by NICE identified several interventions which may reduce exposure to indoor air pollution at home [8]. The cost-effectiveness review identified three US-based economic evaluations, but only one UK-based study, which estimated the cost-effectiveness of enhancing ventilation in the homes of people with asthma [9]. The intervention shifted 17% of children in the intervention group from “severe” to “moderate” asthma, compared with a 3% shift in the control group. The mean cost of these modifications was £1,718 per child treated or £12,300 per child shifted from “severe” to “moderate”. Healthcare costs over 12 months following randomisation did not differ significantly between the intervention and control groups. However, this study was based alongside a randomised controlled trial that did not report any differences in the frequency of asthma exacerbations, nor levels of indoor air pollution, as an outcome measure [9].

England-based economic evaluations are needed to determine the maximum likely amount that could be spent on interventions that would be cost-saving from a National Health Service (NHS) perspective. Therefore, the objective of this study was to design an economic framework that could be used by individual policy decision-makers within England when considering the implementation of interventions designed to reduce indoor air pollution within dwellings. The framework intended to estimate the required level of efficacy necessary, and maximum cost, for a particular intervention to be cost-saving in key settings across certain property types/dwellings and patient groups. The framework focused upon the health conditions associated with exposure to indoor air pollution and was designed to estimate economic outcomes for each condition separately. The framework was intended to be an interactive “calculator” to be made available to those at which the guidance is aimed, who are considering implementing home-based interventions to reduce exposure to indoor air pollution. The framework allowed the user to input values and generate results specific to particular dwelling characteristics on a case-by-case basis.

## 2. Materials and Methods

For detailed information regarding the methods, inputs, modelling assumptions and results please refer to reference [10].

### 2.1. Economic Framework

**Figure 1:**
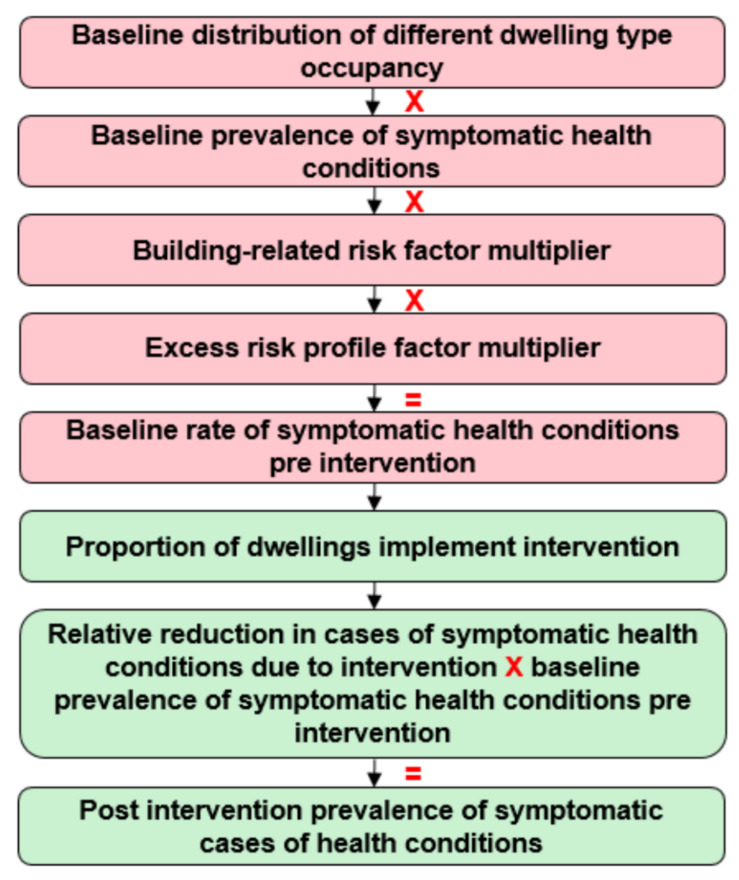
Framework structure.

An economic modelling framework was built in Microsoft Excel to estimate the incremental costs pre- and post-implementation of any interventions designed to reduce exposure to indoor air pollution within dwellings. A schematic of the framework, used to estimate the economic outcomes associated with a reduction in exposure to indoor air pollution, is outlined in Figure 1. The following model flow describes how the number of people with pre-specified health conditions were estimated pre- and post-intervention implementation, over a five-year time horizon:

- The estimated proportion of occupancy in different types of dwellings was stratified based on the reported tenure of homeownership by the English Housing Survey (2016) [11] (Table S1).
- The baseline prevalence of each symptomatic health condition was applied to the population regardless of homeownership modality (Table S2).
- An excess risk of prevalence was applied to each condition, dependent upon physical building, (for example, damp homes), and non-building factors (for example the elderly or people with comorbidities) to estimate the overall baseline prevalence pre-intervention.
- The proportion of dwellings with condition-specific baseline prevalence was then estimated.
- It was assumed that a set proportion of dwellings with the overall risk profile (a combination of baseline risk, building characteristics and non-building characteristics) would implement the intervention.
- An expected relative reduction in symptoms of each health condition was applied to estimate the number of people within the population, post-intervention, that had each condition.
- The costs associated with each condition were then applied to the number of inhabitants with the condition pre- and post-intervention (Tables S2 and S3).
- Furthermore, the upfront and annual costs of the intervention were applied to the dwellings in which it was implemented.

### 2.2. Dwelling Occupancy and Risk Factors

It was assumed that all dwellings in England could be classified by the following types of tenure: owner-occupied, private rented, local authority or housing association. The English Housing Survey was used to determine the total number of dwellings in England and to stratify them by tenure [11]. The average number of inhabitants per dwelling in the United Kingdom [2,3] was then multiplied by the number of dwellings to estimate the total number of inhabitants, across each tenure category, within the baseline population [12] (Supplementary Materials Part I).

The PHAC commented that the building-related risk profile of dwellings could increase the likelihood of exposure to indoor air pollution. One of the following building-related risk factors could be selected by the model user: non-decent homes, usable floor area <90 m^2^ or any damp problems [11]. Data from the English Housing Survey informed the number of dwellings with each building-related risk factor, allowing the number of inhabitants at increased risk of exposure to indoor air pollution to be estimated. A building-related risk factor multiplier (an assumption to be determined on a case-by-case basis) was then applied to the baseline prevalence of each symptomatic health condition associated with patients living in dwellings with the building-related risk factor. It was assumed that the building-related risk factors associated with dwellings were mutually exclusive due to a lack of data available regarding the number of dwellings with multiple factors.

Furthermore, the baseline prevalence of symptomatic health conditions could also be increased by a multiplier based on the presence of an excess risk profile. This was also an assumption-based input which could be determined on a case-by-case basis. This additional excess risk profile was applied multiplicatively to the building-related risk profile and was designed to encompass many other factors that may increase the risk of an inhabitant being exposed to indoor air pollution. Factors that may be incorporated into this risk profile could include, but were not limited to: crowded homes, comorbidities, elderly people, socioeconomic factors, frailty and poverty.

### 2.3. Risk Profile of Homes in England

**Figure 2:**
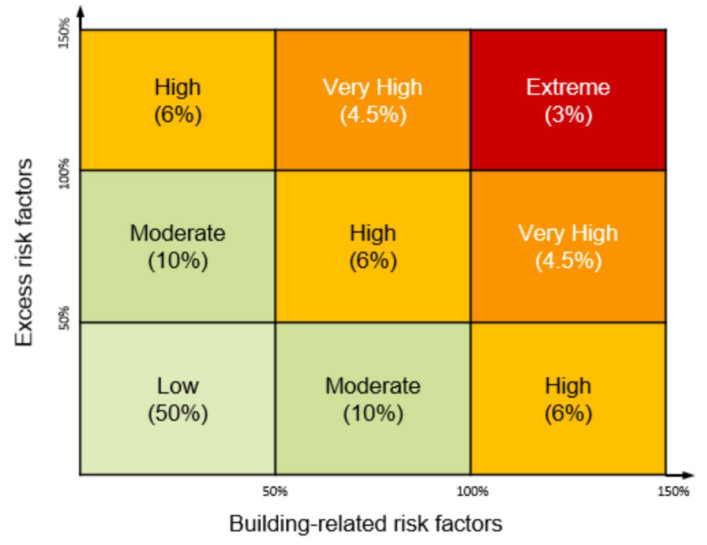
Distribution of dwellings by increased risk factor.

The building-related and excess risk factors were combined to allocate a risk profile to each dwelling within England. It was assumed that whilst the majority of dwellings would have a minimal excess risk profile, for some dwellings the excess risk profile would be very high. The risk profile of the dwelling could be used by decision-makers to determine the type of intervention that would be most applicable. For example, an intervention that would improve the structural properties of the home would be appropriate for a dwelling with a very high building-related risk factor but low excess risk. The assumed distribution of increased risk associated with both excess and building-related risk factors across dwellings in England is represented in Figure 2 (with the mid-point of each box used in the analysis).

### 2.4. Application of Intervention

The framework could be used to estimate the economic outcomes associated with the wide range of interventions that are available to reduce exposure to indoor air pollution and were identified in the review of effectiveness data conducted by NICE (such interventions could range from fitting ventilation systems to encouraging inhabitants to open windows frequently) [8]. Both the costs and efficacy associated with such interventions vary substantially. Therefore, the definition of an intervention was flexible to allow all the economic outcomes of all relevant options to be compared easily.

The upfront intervention cost was varied between £0 and £250 per dwelling within a threshold analysis. The efficacy of the intervention was determined by a relative reduction in the number of symptomatic cases of each health condition associated with the inhabitants of dwellings in which the intervention was implemented. Whilst a wider range was initially considered, the efficacy of the intervention was varied between 0% and 10% for the purposes of this study. This range was considered wide enough to present the maximum price and minimum efficacy at which an intervention is no longer cost-saving under each scenario.

Inhabitants living in dwellings with a building-related risk factor were assumed to have been exposed to higher levels of indoor air pollution at baseline compared with those living in dwellings without a building-related risk factor. Due to the higher baseline risk of indoor air pollution, it was assumed that inhabitants living in dwellings with a building-related risk factor had an increased capacity to benefit from an intervention. Therefore, it was assumed that fewer inhabitants in dwellings without any increased risk would implement an intervention to reduce exposure to indoor air pollution due to an absence of need. For simplicity, it was also assumed that the effectiveness of each intervention remained constant over the five-year time horizon of the model.

In certain circumstances the implementation of an intervention to reduce exposure to indoor air pollution, for example, a ventilation system designed to remove mould and mildew, may be mandated by regulation. Hence, the owners of all dwellings under the regulation would be assumed to adhere to the intervention. However, alternative interventions which cannot be regulated, such as the recommendation for windows to be opened regularly, would have lower adherence rates. Therefore, the framework could be used to estimate the economic outcomes associated with interventions at varied rates of implementation. The cost and efficacy of the intervention were only applied to dwellings that were assumed to have implemented the intervention.

### 2.5. Health Conditions

The health conditions considered most relevant to indoor air exposure were informed by clinical advice from the PHAC. Asthma, COPD and allergic rhinitis were included in the model because they are directly impacted by indoor air pollution. The PHAC also expressed an interest in the effects that exposure to indoor air pollution could have on the mental health of inhabitants. Generalized anxiety disorder (GAD) was also included in the model because it was considered to be an appropriate proxy that incorporated a range of aspects regarding mental health.

The baseline prevalence of each health condition in England, before the implementation of an intervention and before consideration of both the building-related and excess risk factors, was sourced from published literature (Supplementary Materials Part I). It was assumed that baseline prevalence represented symptomatic cases of each health condition.

An annual unit cost was estimated for each health condition and attributed to each predicted symptomatic inhabitant pre- and post-intervention. All unit costs were inflated to the 2017/2018 price year. Further detail regarding the estimation of the unit costs for asthma and COPD is provided within Supplementary Materials Part I. All unit costs were discounted at a five-year discount ratio of 0.935. This discount rate was based on a rate of 3.5% per year in alignment with the NICE guides to the methods of technology appraisal (7). Further information regarding the derivation of this discount rate is presented in Supplementary Materials Part I.

## 3. Results

### 3.1. Presentation of Results

**Figure 3:**
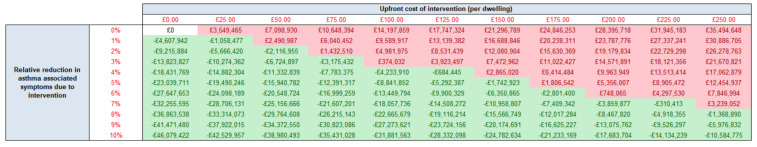
Incremental total cost varying the effectiveness and cost of the intervention across England over five years; extreme risk; 100% implementation rate; non-decent homes (asthma).

**Figure 4:**
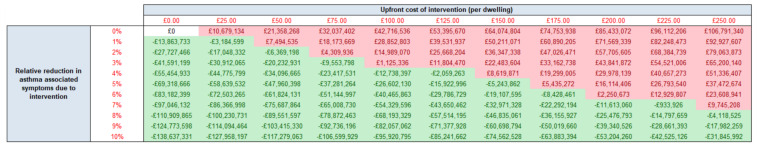
Incremental total cost varying the effectiveness and cost of the intervention across England over five years; extreme risk; 100% implementation rate; usable floor area <90 m^2^ (asthma).

**Figure 5:**
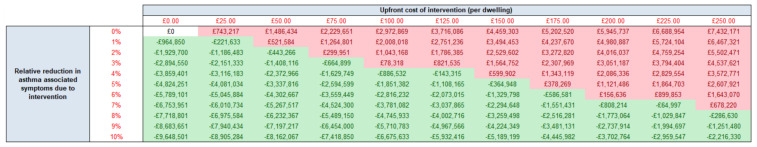
Incremental total cost varying the effectiveness and cost of the intervention across England over five years; extreme risk; 100% implementation rate; any damp problem (asthma).

The results shown in Figure 3, Figure 4 and Figure 5 are presented as sensitivity analyses due to the multiple factors that can determine the economic outcomes of interventions designed to reduce exposure to indoor air pollution. With such a wide range of interventions, it is was considered more useful for a range of inputs to be displayed. Presenting one “fixed result” was not considered appropriate due to difficulty generalizing the framework to all settings. Hence, the results presented cover many different permutations of scenarios, settings and interventions.

Two-way sensitivity analysis is a technique used in economic evaluation to assess the robustness of the overall result when simultaneously varying the values of two key input variables [13]. The upfront intervention cost is varied simultaneously with the potential relative reduction in symptomatic cases of each health condition. The tables allow the user to establish the point at which an intervention is expected to become cost-saving. As aforementioned, these results were based on several assumptions. Therefore, the focus should be on the colourings reflecting cost-savings, and the distance of the parameter combination from the cost-saving threshold, rather than the particular numbers reported.

The plausible ranges of effectiveness were set from 0% to 10% and upfront intervention costs from £0 to £250. All other factors are held constant.

### 3.2. Two-Way Sensitivity Analysis

Figure 3, Figure 4 and Figure 5 present the incremental cost pre- and post-intervention with varying levels of effectiveness and intervention costs for people with asthma, living in extreme risk dwellings that are considered either non-decent, to have a usable floor area <90 m^2^ or to have any damp problems respectively. The results of the analyses demonstrate that at a given level of effectiveness, the threshold for the upfront cost of the intervention to remain cost-saving is equivalent across the different home characteristics. For example, an intervention that costs £50 must lead to a minimum relative reduction in cases of symptomatic asthma of 2% in each extreme risk dwelling to be cost-saving. Out of the three building-related risk factors, dwellings with a usable floor area <90 m^2^ have the greatest capacity to benefit, with the largest cost-saving, at any given level of effectiveness and upfront cost. When an intervention costs £50 and leads to a 2% relative reduction in cases of symptomatic asthma, cost-savings for dwellings with a useable floor area <90 m^2^ were £6,369,198 compared with £2,116,955 and £443,266 for non-decent or damp homes, respectively.

Two-way sensitivity analysis presenting the incremental cost pre- and post-intervention for extreme and low risk households (assuming a 100% and 50% implementation rate, respectively), across all four health conditions are presented in Supplementary Materials Part II. For conditions other than asthma, no intervention was cost-saving if it cost more than £200 per dwelling. As anticipated, the figures suggest that households with an extreme risk profile have a greater capacity to benefit from interventions designed to reduce exposure to indoor air pollution than low risk households. The highest potential savings could all be made in homes with usable floor area <90 m^2^. If a free intervention could reduce asthma symptoms by 10%, with 50% implementation, this could generate £356,577,498 over five years in England; for GAD, the same conditions would generate £239,556,803; for allergic rhinitis, £222,842,005; and for COPD, £165,212,635. The relationship between the risk profile and capacity to benefit follows the same trend across all four health conditions. However, due to the condition having the lowest prevalence, the effectiveness of the intervention was most important when considering the cost-savings for symptomatic COPD cases. For example, an intervention that is 10% effective and costing £175 would be cost-saving when used to reduce cases of asthma, GAD and allergic rhinitis in an extreme risk dwelling, but not COPD. The results show that for dwellings with a low risk profile, the stakeholder can be reasonably certain than an intervention priced at £100 is unlikely to result in cost-savings if it does not reduce symptomatic cases of asthma by 10% or more. However, in an extreme risk dwelling, the stakeholder can be reasonably certain that an intervention priced at £100 will result in cost-savings at a minimum reduction in symptomatic cases of asthma of 4%.

## 4. Discussion

As far as the authors are aware, this is the first economic model estimating the cost-savings associated with the implementation of multiple interventions designed to reduce indoor air pollution in England. The model is very flexible and can be used to guide decision-making under a wide range of scenarios. The model is predicated on a high level of assumptions around many key variables. This notwithstanding, the results show that key drivers of the cost difference include the excess risk profile dwellings, upfront cost and the effectiveness of the intervention designed to reduce exposure to indoor air pollution.

Across all of the clinical outcomes and dwelling types, a key finding from our work is that the cost of intervention can be higher as the risk profile of a dwelling is increased, at a given level of efficacy, before it is no longer cost-saving. However, this needs to be considered in line with the results from the US study, where one of the major factors affecting program cost was the level of intensity of environmental remediation (minor, moderate or major) [5], meaning it was more costly to improve conditions in high risk dwellings.

Similarly, and regardless of the dwelling building characteristic, the cost of an intervention can be higher and still be cost-saving for a dwelling with an extreme risk profile compared with a low risk profile for a given level of efficacy. For example, an intervention with a relative reduction in cases of symptomatic cases of asthma of 5%, must cost a maximum of £50 per household to be considered cost-saving in a “non-decent” dwelling with a low risk profile (Supplementary Materials Part II). However, the intervention cost can cost up to £150 per “non-decent” dwelling with an extreme risk profile and still be cost-saving (Figure 3). This type of threshold analysis could help a stakeholder decide whether to implement an intervention to reduce exposure to indoor air pollution. The results of this study are in alignment with a published cost-effectiveness analysis of enhanced ventilation within the homes of children with asthma. The incremental cost per unit improvement in PedsQL points (The Pediatric Quality of Life Inventory) was £165 and £379 for children with severe and moderate asthma, respectively [9].

It is difficult to compare the results from this model with previous studies as this is the only study known to provide a range of intervention costs and the likely cost-saving in relation to intervention effectiveness. However, when compared with the US asthma study, where program costs per participant per year ranged from $231 to $14,858 (in 2007 US$), savings were estimated to be between 5.3 and 14 times the intervention [5]. For example, the program costs of reducing asthma triggers in homes of children reported by Oatman was $497, but the direct medical costs averted were $2637 [14]. Cost-savings were higher in the 2005 study from Shelledy, where program costs for paediatric asthma disease management were $721 but medical cost-savings were $10,093 [15]. This would correspond with the savings seen for asthma within extreme risk non-decent homes (with approximately 146,636 people in England meeting this criterion). If an intervention led to a relative reduction of cases by 2% and had an upfront intervention cost of £50, this would lead to an approximate cost-saving of £14.44 per person within this population.

It was assumed that, because of the substantial health risks associated with indoor air pollution, there would be a 100% implementation rate in extreme risk dwellings. However, the implementation rate may be as low as 50% in low risk dwellings due to the lack of necessity. All other factors remaining equal, a lower implementation rate led to a smaller capacity to benefit, and a smaller reduction in overall cost savings.

Due to a lack of data availability, the baseline prevalence of each symptomatic health condition was independent of the risk profile of dwellings and assumed to be equivalent across all risk profiles. Therefore, the cost-savings per person associated with the elimination of health symptoms are equivalent regardless of their risk profile. The study authors anticipated that the majority of dwellings would have a minimal excess risk profile and a very low number of dwellings would have a very high excess risk profile. Therefore, although there is greater capacity for people at high risk to benefit from interventions that reduce exposure to indoor air pollution within homes, greater overall cost-savings accrue for those at low risk due to a higher number of people benefiting from the intervention.

The method of presenting model results using a “what-if?” sensitivity analysis was deemed to be the most appropriate based on the available data and the nature of the interventions to be assessed. However, since the model is designed to be a framework to guide decision-makers (national and local) in allocating resources, the inputs in the model are intended as a starting point for discussion and to give a general overview of the direction of results. The use of sensitivity analyses allows the model results to be relevant to a wide group of stakeholders. The model is designed as an interactive “calculator” which is intended to be used flexibly so that it can be tailored to a particular dwelling with a specific risk profile and a specific intervention. The risk profile of a dwelling should be used by decision-makers to determine the type of intervention that would be most applicable to the particular dwelling. For example, an intervention designed to improve the structural properties of the home would be more appropriate than an intervention to improve heating efficacy for dwellings with a very high building-related risk factor but low excess risk factor (e.g., an affluent family living in a home with damp).

### Limitations

Although not modelled explicitly, the link between indoor air quality and health has been captured implicitly through the relationship between the increased likelihood of exposure to indoor air pollution, the risk profile of each dwelling and the health outcomes included in the model.

Many inhabitants with exposure to indoor air pollution will likely have more than one symptomatic health condition simultaneously. It would be expected that, if a person had comorbidities, an intervention might impact several health conditions rather than one alone. Unfortunately, the model does not include the functionality to account for these comorbidities as it was not possible to estimate the comorbidity status of each inhabitant in the population. Therefore, the economic model may underestimate the true level of cost-savings to the NHS.

It was also assumed that the building-related risk factors associated with dwellings are mutually exclusive due to the lack of data available regarding the likelihood of a dwelling having an additional building-related risk profile. However, it is unrealistic to assume a dwelling considered “non-decent” would not have “any damp problems” or a usable floor area greater than <90 m^2^. Due to the level of complexity required, the economic model also does not estimate the cost-effectiveness of multiple interventions applied within a dwelling simultaneously. It is likely, that by separating the three building-related risk profiles, that the number of dwellings, and subsequently inhabitants captured within each analysis is underestimated. Furthermore, an intervention may reduce the risk associated with more than one building-related risk factor. Therefore, the economic model may further underestimate the true level of cost-savings associated with an intervention.

Due to the complexity of modelling that would be required, it was assumed that inhabitants do not move dwellings throughout the five-year time horizon of the model. If inhabitants move out of a home that has a non-transferable intervention, for example, a ventilation system that would be too costly to remove, they will no longer benefit from a reduction in indoor air pollution and the cost savings to the NHS within the model may be overestimated. It was also assumed that, with the exception of the intervention, no other improvements would be made to the home to reduce exposure to indoor air pollution, nor any changes to building regulations made. If improvements had been made, the baseline prevalence of symptomatic conditions would be lower and the health improvements due to the intervention overestimated in the model.

Furthermore, the model only estimates the potential cost savings of interventions to reduce exposure to indoor air pollution at home from the perspective of the NHS. The inclusion of any non-NHS benefits from other perspectives, both financially and non-financially, would have only improved the cost-effectiveness of interventions.

## 5. Conclusions

The implementation of interventions designed to reduce exposure to indoor air pollutants has the potential to reduce symptomatic health conditions such as asthma, COPD, GAD and allergic rhinitis. The capacity to benefit increases as the risk profile of a dwelling increases and the key drivers of savings are the excess risk profile of dwellings and the upfront cost and effectiveness of the specified intervention. The likelihood of an intervention being cost-saving is highest when considering asthma, and lowest for COPD, reflecting the high prevalence of asthma and low prevalence of COPD. The model framework can be used by individual policy decision-makers when considering the implementation of interventions designed to reduce indoor air pollution within dwellings to estimate the required level of efficacy necessary for a particular intervention to be cost-saving.

## Data Availability

Data sharing not applicable.

## Supplementary Materials

The following are available online at https://www.mdpi.com/1660-4601/18/4/1679/s1, Figure S1: Incremental total cost varying the effectiveness and cost of the intervention across England over five years; low risk; 50% implementation rate; non-decent homes (asthma), Figure S2: Incremental total cost varying the effectiveness and cost of the intervention across England over five years; low risk; 50% implementation rate; usable floor area <90 m^2^ (asthma), Figure S3: Incremental total cost varying the effectiveness and cost of the intervention across England over five years; low risk; 50% implementation rate; any damp problem (asthma), Figure S4: Incremental total cost varying the effectiveness and cost of the intervention across England over five years; extreme risk; 100% implementation rate; non-decent homes (COPD), Figure S5: Incremental total cost varying the effectiveness and cost of the intervention across England over five years; extreme risk; 100% implementation rate; usable floor area <90 m^2^ (COPD), Figure S6: Incremental total cost varying the effectiveness and cost of the intervention across England over five years; extreme risk; 100% implementation rate; any damp problem (COPD), Figure S7: Incremental total cost varying the effectiveness and cost of the intervention across England over five years; low risk; 50% implementation rate; non-decent homes (COPD), Figure S8: Incremental total cost varying the effectiveness and cost of the intervention across England over five years; low risk; 50% implementation rate; usable floor area <90 m^2^ (COPD), Figure S9: Incremental total cost varying the effectiveness and cost of the intervention across England over five years; low risk; 50% implementation rate; any damp problem (COPD), Figure S10: Incremental total cost varying the effectiveness and cost of the intervention across England over five years; extreme risk; 100% implementation rate; non-decent homes (allergic rhinitis), Figure S11: Incremental total cost varying the effectiveness and cost of the intervention across England over five years; extreme risk; 100% implementation rate; usable floor area <90 m^2^ (allergic rhinitis), Figure S12: Incremental total cost varying the effectiveness and cost of the intervention across England over five years; extreme risk; 100% implementation rate; any damp problem (allergic rhinitis), Figure S13: Incremental total cost varying the effectiveness and cost of the intervention across England over five years; low risk; 50% implementation rate; non-decent homes (allergic rhinitis), Figure S14: Incremental total cost varying the effectiveness and cost of the intervention across England over five years; low risk; 50% implementation rate; usable floor area <90 m^2^ (allergic rhinitis), Figure S15: Incremental total cost varying the effectiveness and cost of the intervention across England over five years; low risk; 50% implementation rate; any damp problems (allergic rhinitis), Figure S16: Incremental total cost varying the effectiveness and cost of the intervention across England over five years; extreme risk; 100% implementation rate; non-decent homes (GAD), Figure S17: Incremental total cost varying the effectiveness and cost of the intervention across England over five years; extreme risk; 100% implementation rate; usable floor area <90 m^2^ (GAD), Figure S18: Incremental total cost varying the effectiveness and cost of the intervention across England over five years; extreme risk; 100% implementation rate; any damp problem (GAD), Figure S19: Incremental total cost varying the effectiveness and cost of the intervention across England over five years; low risk; 50% implementation rate; non-decent homes (GAD), Figure S20: Incremental total cost varying the effectiveness and cost of the intervention across England over five years; low risk; 50% implementation rate; usable floor area <90 m^2^ (GAD), Figure S21: Incremental total cost varying the effectiveness and cost of the intervention across England over five years; low risk; 50% implementation rate; any damp problem (GAD), Table S1: Dwelling occupancy by tenure (2017), Table S2: Health condition related model inputs, Table S3: Derivation of the five-year discount rate.
