# Supplementary material for: Indoor Air Quality at Home—An Economic Analysis
Source: Int J Environ Res Public Health. 2021 Feb 9;18(4):1679. doi: 10.3390/ijerph18041679 (PMC7916172; doi:10.3390/ijerph18041679)
Supplement: Supplementary file 1 [file ijerph-18-01679-s001.pdf]

Article

Indoor Air Quality at Home – An Economic Analysis

Amy Dymond[1], Stuart Mealing[1], Jessica McMaster[1], Hayden Holmes[1], Lesley Owen[2]*

[1] York Health Economics Consortium, Enterprise House, Innovation Way, University of York, Heslington, York, United Kingdom, YO10 5NQ
[2] National Institute for Health and Care Excellence, 10 Spring Gardens, London, United Kingdom, SW1A 2BU
* Correspondence: amy.dymond@york.ac.uk; Tel.: +44 (0)1904 322789

Supplementary material

Appendix A: Model Inputs

Table S.1: Dwelling occupancy by tenure (2017)

Tenure	Distribution of dwellings	Number of dwellings	Total number of inhabitants
Owner-occupied	62.4%	14,809,164	34,061,078
Private rented	20.5%	4,865,190	11,189,937
Local authority	6.8%	1,613,819	3,711,784
Housing association	10.3%	2,444,461	5,622,261
**Total**	**100%**	**23,732,635**	**54,585,061**
**Tenure**	**Non-decent homes**	**Usable floor area <90m2**	**Any damp problem**
Owner-occupied	19.7%	46.7%	2.7%
Private rented	26.8%	75.8%	8.2%
Local authority	12.6%	91.2%	6.0%
Housing association	12.6%	88.5%	3.9%

Table S.2: Health condition related model inputs

Condition	Prevalence	Source
Asthma	8.9%	[10]
COPD	2.0%	[11]
Allergic rhinitis	10%	[12]
GAD	4.4%	[13]
**Condition**	**Annual cost**	**Source**
Asthma	£672	[14, 15]
COPD	£1,382	[16]
Allergic rhinitis	£373	[17]
GAD	£911	[13]

Abbreviations: COPD, continuous obstructive pulmonary disease; GAD, generalized anxiety disorder

Table S.3: Cost inputs (asthma and COPD)

Health state (asthma)	Unit costs (weekly)	Exacerbations	Duration of exacerbation (week)	Weeks spent in health state	Source
Controlled	£12.23	N/A	N/A	51.38	[14, 15]
Uncontrolled	£46.61	N/A	N/A	0.50	
Moderate exacerbation	£72.57	0.10	1	0.10	
Severe exacerbation	£669.95	0.01	2	0.02	
**Disease severity (COPD)**	**Prevalence**	**Monthly cost**	**Non-severe exacerbation rate (monthly)**	**Severe exacerbation rate (monthly)**	**Source**
Mild	17.1%	£43.15	0.05	0.01	[16, 18]
Moderate	52.2%	£43.15	0.05	0.01	
Severe	25.5%	£90.55	0.06	0.02	
Very severe	5.2%	£149.53	0.07	0.03	

The weekly cost of asthma was sourced from a NICE technology appraisal for eosinophilic asthma (TA479) [14]. No data were identified on the number of weeks per year an individual with asthma will spend with symptoms of varying severity. The duration of asthma of a severe or moderate exacerbation was sourced from TA479. The average annual number of exacerbations an individual with asthma will have (0.11) was sourced from a retrospective cohort study describing asthma exacerbation frequency and risk [15]. However, this study did not inform the distributional split of these exacerbations between moderate or severe. Therefore, it was assumed that the majority of exacerbations would be moderate (0.1) and only a small proportion severe (0.01). It was assumed that patients would spend 0.5 weeks a year with uncontrolled asthma and would have controlled asthma for the remainder of the year. The number of weeks spent in each health state, per year, was multiplied by the weekly unit cost to generate an annual unit cost per person with asthma of £672 (Table A1).

The proportion of people with COPD at each level of disease severity was sourced from a study informed by a large database of primary care COPD patients in the UK [19]. The monthly costs associated with the treatment of severe and non-severe exacerbations were £4,071 and £129 respectively. These costs were extracted from a cost-utility study of COPD in the UK [16], and were

multiplied by 12 to generate annual costs. A weighted average of these costs, based on the distribution of severity across patients with COPD, was calculated to generate an annual unit cost per person of £1,382 (Table A1). Due to a lack of alternative information, it was assumed that the monthly cost would be equivalent for people with mild and moderate COPD. Similarly, the non-severe and severe exacerbation rates were assumed to be equivalent between those with mild and moderate COPD.

Table S.3: Derivation of five-year discount rate

	**Year 1**	**Year 2**	**Year 3**	**Year 4**	**Year 5**	**Total**
No discounting	1.00000	1.00000	1.00000	1.00000	1.00000	**5.00000**
Discounting	1.00000	0.96618	0.93351	0.90194	0.87144	**4.67308**
**Ratio**						**0.93462**

Appendix B: Two-way sensitivity analysis

Asthma

		Upfront cost of intervention (per dwelling)										
		£0.00	£25.00	£50.00	£75.00	£100.00	£125.00	£150.00	£175.00	£200.00	£225.00	£250.00
Relative reduction in asthma associated symptoms due to intervention	0%	£0	£29,578,873	£59,157,746	£88,736,619	£118,315,492	£147,894,365	£177,473,238	£207,052,111	£236,630,984	£266,209,857	£295,788,730
	1%	-£11,851,703	£17,727,170	£47,306,043	£76,884,916	£106,463,789	£136,042,662	£165,621,535	£195,200,408	£224,779,281	£254,358,154	£283,937,027
	2%	-£23,703,406	£5,875,467	£35,454,340	£65,033,212	£94,612,085	£124,190,958	£153,769,831	£183,348,704	£212,927,577	£242,506,450	£272,085,323
	3%	-£35,555,110	-£5,976,237	£23,602,636	£53,181,509	£82,760,382	£112,339,255	£141,918,128	£171,497,001	£201,075,874	£230,654,747	£260,233,620
	4%	-£47,406,813	-£17,827,940	£11,750,933	£41,329,806	£70,908,679	£100,487,552	£130,066,425	£159,645,298	£189,224,171	£218,803,044	£248,381,917
	5%	-£59,258,516	-£29,679,643	-£100,770	£29,478,103	£59,056,976	£88,635,849	£118,214,722	£147,793,595	£177,372,468	£206,951,341	£236,530,214
	6%	-£71,110,219	-£41,531,346	-£11,952,473	£17,626,400	£47,205,273	£76,784,146	£106,363,019	£135,941,892	£165,520,765	£195,099,637	£224,678,510
	7%	-£82,961,922	-£53,383,050	-£23,804,177	£5,774,696	£35,353,569	£64,932,442	£94,511,315	£124,090,188	£153,669,061	£183,247,934	£212,826,807
	8%	-£94,813,626	-£65,234,753	-£35,655,880	-£6,077,007	£23,501,866	£53,080,739	£82,659,612	£112,238,485	£141,817,358	£171,396,231	£200,975,104
	9%	-£106,665,329	-£77,086,456	-£47,507,583	-£17,928,710	£11,650,163	£41,229,036	£70,807,909	£100,386,782	£129,965,655	£159,544,528	£189,123,401
	10%	-£118,517,032	-£88,938,159	-£59,359,286	-£29,780,413	-£201,540	£29,377,333	£58,956,206	£88,535,079	£118,113,952	£147,692,825	£177,271,698

Figure S.1: Incremental total cost varying the effectiveness and cost of the intervention across England over five years; low risk; 50% implementation rate; non-decent homes (asthma)

		Upfront cost of intervention (per dwelling)										
		£0.00	£25.00	£50.00	£75.00	£100.00	£125.00	£150.00	£175.00	£200.00	£225.00	£250.00
Relative reduction in asthma associated symptoms due to intervention	0%	£0	£88,992,783	£177,985,566	£266,978,349	£355,971,132	£444,963,915	£533,956,698	£622,949,481	£711,942,264	£800,935,047	£889,927,831
	1%	-£35,657,750	£53,335,033	£142,327,816	£231,320,599	£320,313,382	£409,306,165	£498,298,948	£587,291,732	£676,284,515	£765,277,298	£854,270,081
	2%	-£71,315,500	£17,677,283	£106,670,066	£195,662,849	£284,655,633	£373,648,416	£462,641,199	£551,633,982	£640,626,765	£729,619,548	£818,612,331
	3%	-£106,973,250	-£17,980,466	£71,012,317	£160,005,100	£248,997,883	£337,990,666	£426,983,449	£515,976,232	£604,969,015	£693,961,798	£782,954,581
	4%	-£142,630,999	-£53,638,216	£35,354,567	£124,347,350	£213,340,133	£302,332,916	£391,325,699	£480,318,482	£569,311,265	£658,304,048	£747,296,831
	5%	-£178,288,749	-£89,295,966	-£303,183	£88,689,600	£177,682,383	£266,675,166	£355,667,949	£444,660,732	£533,653,515	£622,646,298	£711,639,081
	6%	-£213,946,499	-£124,953,716	-£35,960,933	£53,031,850	£142,024,633	£231,017,416	£320,010,199	£409,002,982	£497,995,765	£586,988,548	£675,981,332
	7%	-£249,604,249	-£160,611,466	-£71,618,683	£17,374,100	£106,366,883	£195,359,666	£284,352,449	£373,345,233	£462,338,016	£551,330,799	£640,323,582
	8%	-£285,261,999	-£196,269,216	-£107,276,433	-£18,283,650	£70,709,134	£159,701,917	£248,694,700	£337,687,483	£426,680,266	£515,673,049	£604,665,832
	9%	-£320,919,749	-£231,926,965	-£142,934,182	-£53,941,399	£35,051,384	£124,044,167	£213,036,950	£302,029,733	£391,022,516	£480,015,299	£569,008,082
	10%	-£356,577,498	-£267,584,715	-£178,591,932	-£89,599,149	-£606,366	£88,386,417	£177,379,200	£266,371,983	£355,364,766	£444,357,549	£533,350,332

Figure S.2: Incremental total cost varying the effectiveness and cost of the intervention across England over five years; low risk; 50% implementation rate; usable floor area <90m^2 (asthma)

		Upfront cost of intervention (per dwelling)										
		£0.00	£25.00	£50.00	£75.00	£100.00	£125.00	£150.00	£175.00	£200.00	£225.00	£250.00
Relative reduction in asthma associated symptoms due to intervention	0%	£0	£6,193,476	£12,386,952	£18,580,428	£24,773,904	£30,967,380	£37,160,857	£43,354,333	£49,547,809	£55,741,285	£61,934,761
	1%	-£2,481,610	£3,711,866	£9,905,342	£16,098,818	£22,292,294	£28,485,770	£34,679,246	£40,872,722	£47,066,198	£53,259,674	£59,453,150
	2%	-£4,963,221	£1,230,255	£7,423,731	£13,617,207	£19,810,683	£26,004,160	£32,197,636	£38,391,112	£44,584,588	£50,778,064	£56,971,540
	3%	-£7,444,831	-£1,251,355	£4,942,121	£11,135,597	£17,329,073	£23,522,549	£29,716,025	£35,909,501	£42,102,977	£48,296,453	£54,489,930
	4%	-£9,926,442	-£3,732,966	£2,460,510	£8,653,986	£14,847,463	£21,040,939	£27,234,415	£33,427,891	£39,621,367	£45,814,843	£52,008,319
	5%	-£12,408,052	-£6,214,576	-£21,100	£6,172,376	£12,365,852	£18,559,328	£24,752,804	£30,946,280	£37,139,756	£43,333,233	£49,526,709
	6%	-£14,889,663	-£8,696,187	-£2,502,711	£3,690,766	£9,884,242	£16,077,718	£22,271,194	£28,464,670	£34,658,146	£40,851,622	£47,045,098
	7%	-£17,371,273	-£11,177,797	-£4,984,321	£1,209,155	£7,402,631	£13,596,107	£19,789,583	£25,983,059	£32,176,536	£38,370,012	£44,563,488
	8%	-£19,852,884	-£13,659,408	-£7,465,931	-£1,272,455	£4,921,021	£11,114,497	£17,307,973	£23,501,449	£29,694,925	£35,888,401	£42,081,877
	9%	-£22,334,494	-£16,141,018	-£9,947,542	-£3,754,066	£2,439,410	£8,632,886	£14,826,362	£21,019,839	£27,213,315	£33,406,791	£39,600,267
	10%	-£24,816,105	-£18,622,628	-£12,429,152	-£6,235,676	-£42,200	£6,151,276	£12,344,752	£18,538,228	£24,731,704	£30,925,180	£37,118,656

Figure S.3: Incremental total cost varying the effectiveness and cost of the intervention across England over five years; low risk; 50% implementation rate; any damp problem (asthma)

COPD

		Upfront cost of intervention (per dwelling)										
		£0.00	£25.00	£50.00	£75.00	£100.00	£125.00	£150.00	£175.00	£200.00	£225.00	£250.00
Relative reduction in chronic obstructive pulmonary disease associated symptoms due to intervention	0%	£0	£3,549,465	£7,098,930	£10,648,394	£14,197,859	£17,747,324	£21,296,789	£24,846,253	£28,395,718	£31,945,183	£35,494,648
	1%	-£2,134,992	£1,414,472	£4,963,937	£8,513,402	£12,062,867	£15,612,331	£19,161,796	£22,711,261	£26,260,726	£29,810,190	£33,359,655
	2%	-£4,269,985	-£720,520	£2,828,945	£6,378,409	£9,927,874	£13,477,339	£17,026,804	£20,576,268	£24,125,733	£27,675,198	£31,224,663
	3%	-£6,404,977	-£2,855,513	£693,952	£4,243,417	£7,792,882	£11,342,346	£14,891,811	£18,441,276	£21,990,741	£25,540,205	£29,089,670
	4%	-£8,539,970	-£4,990,505	-£1,441,040	£2,108,424	£5,657,889	£9,207,354	£12,756,819	£16,306,283	£19,855,748	£23,405,213	£26,954,678
	5%	-£10,674,962	-£7,125,498	-£3,576,033	-£26,568	£3,522,897	£7,072,361	£10,621,826	£14,171,291	£17,720,756	£21,270,220	£24,819,685
	6%	-£12,809,955	-£9,260,490	-£5,711,025	-£2,161,561	£1,387,904	£4,937,369	£8,486,834	£12,036,298	£15,585,763	£19,135,228	£22,684,693
	7%	-£14,944,947	-£11,395,483	-£7,846,018	-£4,296,553	-£747,088	£2,802,376	£6,351,841	£9,901,306	£13,450,771	£17,000,236	£20,549,700
	8%	-£17,079,940	-£13,530,475	-£9,981,010	-£6,431,546	-£2,882,081	£667,384	£4,216,849	£7,766,314	£11,315,778	£14,865,243	£18,414,708
	9%	-£19,214,932	-£15,665,468	-£12,116,003	-£8,566,538	-£5,017,073	-£1,467,608	£2,081,856	£5,631,321	£9,180,786	£12,730,251	£16,279,715
	10%	-£21,349,925	-£17,800,460	-£14,250,995	-£10,701,530	-£7,152,066	-£3,602,601	-£53,136	£3,496,329	£7,045,793	£10,595,258	£14,144,723

Figure S.4: Incremental total cost varying the effectiveness and cost of the intervention across England over five years; extreme risk; 100% implementation rate; non-decent homes (COPD)

		Upfront cost of intervention (per dwelling)										
		£0.00	£25.00	£50.00	£75.00	£100.00	£125.00	£150.00	£175.00	£200.00	£225.00	£250.00
Relative reduction in chronic obstructive pulmonary disease associated symptoms due to intervention	0%	£0	£10,679,134	£21,358,268	£32,037,402	£42,716,536	£53,395,670	£64,074,804	£74,753,938	£85,433,072	£96,112,206	£106,791,340
	1%	-£6,423,467	£4,255,667	£14,934,801	£25,613,935	£36,293,069	£46,972,203	£57,651,337	£68,330,471	£79,009,604	£89,688,738	£100,367,872
	2%	-£12,846,935	-£2,167,801	£8,511,333	£19,190,467	£29,869,601	£40,548,735	£51,227,869	£61,907,003	£72,586,137	£83,265,271	£93,944,405
	3%	-£19,270,402	-£8,591,268	£2,087,866	£12,767,000	£23,446,134	£34,125,268	£44,804,402	£55,483,536	£66,162,670	£76,841,804	£87,520,938
	4%	-£25,693,869	-£15,014,735	-£4,335,601	£6,343,533	£17,022,667	£27,701,801	£38,380,935	£49,060,069	£59,739,203	£70,418,337	£81,097,471
	5%	-£32,117,336	-£21,438,202	-£10,759,068	-£79,934	£10,599,200	£21,278,334	£31,957,468	£42,636,601	£53,315,735	£63,994,869	£74,674,003
	6%	-£38,540,804	-£27,861,670	-£17,182,536	-£6,503,402	£4,175,732	£14,854,866	£25,534,000	£36,213,134	£46,892,268	£57,571,402	£68,250,536
	7%	-£44,964,271	-£34,285,137	-£23,606,003	-£12,926,869	-£2,247,735	£8,431,399	£19,110,533	£29,789,667	£40,468,801	£51,147,935	£61,827,069
	8%	-£51,387,738	-£40,708,604	-£30,029,470	-£19,350,336	-£8,671,202	£2,007,932	£12,687,066	£23,366,200	£34,045,334	£44,724,468	£55,403,602
	9%	-£57,811,205	-£47,132,071	-£36,452,937	-£25,773,803	-£15,094,669	-£4,415,535	£6,263,599	£16,942,732	£27,621,866	£38,301,000	£48,980,134
	10%	-£64,234,673	-£53,555,539	-£42,876,405	-£32,197,271	-£21,518,137	-£10,839,003	-£159,869	£10,519,265	£21,198,399	£31,877,533	£42,556,667

Figure S.5: Incremental total cost varying the effectiveness and cost of the intervention across England over five years; extreme risk; 100% implementation rate; usable floor area <90m² (COPD)

		Upfront cost of intervention (per dwelling)										
		£0.00	£25.00	£50.00	£75.00	£100.00	£125.00	£150.00	£175.00	£200.00	£225.00	£250.00
Relative reduction in chronic obstructive pulmonary disease associated symptoms due to intervention	0%	£0	£743,217	£1,486,434	£2,229,651	£2,972,869	£3,716,086	£4,459,303	£5,202,520	£5,945,737	£6,688,954	£7,432,171
	1%	-£447,043	£296,174	£1,039,391	£1,782,609	£2,525,826	£3,269,043	£4,012,260	£4,755,477	£5,498,694	£6,241,911	£6,985,128
	2%	-£894,086	-£150,869	£592,348	£1,335,566	£2,078,783	£2,822,000	£3,565,217	£4,308,434	£5,051,651	£5,794,868	£6,538,086
	3%	-£1,341,129	-£597,912	£145,306	£888,523	£1,631,740	£2,374,957	£3,118,174	£3,861,391	£4,604,608	£5,347,826	£6,091,043
	4%	-£1,788,172	-£1,044,954	-£301,737	£441,480	£1,184,697	£1,927,914	£2,671,131	£3,414,348	£4,157,565	£4,900,783	£5,644,000
	5%	-£2,235,214	-£1,491,997	-£748,780	-£5,563	£737,654	£1,480,871	£2,224,088	£2,967,305	£3,710,523	£4,453,740	£5,196,957
	6%	-£2,682,257	-£1,939,040	-£1,195,823	-£452,606	£290,611	£1,033,828	£1,777,045	£2,520,263	£3,263,480	£4,006,697	£4,749,914
	7%	-£3,129,300	-£2,386,083	-£1,642,866	-£899,649	-£156,432	£586,785	£1,330,003	£2,073,220	£2,816,437	£3,559,654	£4,302,871
	8%	-£3,576,343	-£2,833,126	-£2,089,909	-£1,346,692	-£603,475	£139,743	£882,960	£1,626,177	£2,369,394	£3,112,611	£3,855,828
	9%	-£4,023,386	-£3,280,169	-£2,536,952	-£1,793,735	-£1,050,517	-£307,300	£435,917	£1,179,134	£1,922,351	£2,665,568	£3,408,785
	10%	-£4,470,429	-£3,727,212	-£2,983,995	-£2,240,777	-£1,497,560	-£754,343	-£11,126	£732,091	£1,475,308	£2,218,525	£2,961,742

Figure S.61: Incremental total cost varying the effectiveness and cost of the intervention across England over five years; extreme risk; 100% implementation rate; any damp problem (COPD)

		Upfront cost of intervention (per dwelling)										
		£0.00	£25.00	£50.00	£75.00	£100.00	£125.00	£150.00	£175.00	£200.00	£225.00	£250.00
Relative reduction in chronic obstructive pulmonary disease associated symptoms due to intervention	0%	£0	£29,578,873	£59,157,746	£88,736,619	£118,315,492	£147,894,365	£177,473,238	£207,052,111	£236,630,984	£266,209,857	£295,788,730
	1%	-£5,491,236	£24,087,637	£53,666,510	£83,245,383	£112,824,256	£142,403,129	£171,982,002	£201,560,875	£231,139,748	£260,718,621	£290,297,494
	2%	-£10,982,472	£18,596,401	£48,175,274	£77,754,147	£107,333,020	£136,911,893	£166,490,766	£196,069,639	£225,648,512	£255,227,385	£284,806,258
	3%	-£16,473,707	£13,105,166	£42,684,039	£72,262,912	£101,841,785	£131,420,658	£160,999,530	£190,578,403	£220,157,276	£249,736,149	£279,315,022
	4%	-£21,964,943	£7,613,930	£37,192,803	£66,771,676	£96,350,549	£125,929,422	£155,508,295	£185,087,168	£214,666,041	£244,244,914	£273,823,787
	5%	-£27,456,179	£2,122,694	£31,701,567	£61,280,440	£90,859,313	£120,438,186	£150,017,059	£179,595,932	£209,174,805	£238,753,678	£268,332,551
	6%	-£32,947,415	-£3,368,542	£26,210,331	£55,789,204	£85,368,077	£114,946,950	£144,525,823	£174,104,696	£203,683,569	£233,262,442	£262,841,315
	7%	-£38,438,650	-£8,859,778	£20,719,095	£50,297,968	£79,876,841	£109,455,714	£139,034,587	£168,613,460	£198,192,333	£227,771,206	£257,350,079
	8%	-£43,929,886	-£14,351,013	£15,227,860	£44,806,733	£74,385,606	£103,964,479	£133,543,352	£163,122,225	£192,701,098	£222,279,970	£251,858,843
	9%	-£49,421,122	-£19,842,249	£9,736,624	£39,315,497	£68,894,370	£98,473,243	£128,052,116	£157,630,989	£187,209,862	£216,788,735	£246,367,608
	10%	-£54,912,358	-£25,333,485	£4,245,388	£33,824,261	£63,403,134	£92,982,007	£122,560,880	£152,139,753	£181,718,626	£211,297,499	£240,876,372

Figure S.7: Incremental total cost varying the effectiveness and cost of the intervention across England over five years; low risk; 50% implementation rate; non-decent homes (COPD)

		Upfront cost of intervention (per dwelling)										
		£0.00	£25.00	£50.00	£75.00	£100.00	£125.00	£150.00	£175.00	£200.00	£225.00	£250.00
Relative reduction in chronic obstructive pulmonary disease associated symptoms due to intervention	0%	£0	£88,992,783	£177,985,566	£266,978,349	£355,971,132	£444,963,915	£533,956,698	£622,949,481	£711,942,264	£800,935,047	£889,927,831
	1%	-£16,521,264	£72,471,520	£161,464,303	£250,457,086	£339,449,869	£428,442,652	£517,435,435	£606,428,218	£695,421,001	£784,413,784	£873,406,567
	2%	-£33,042,527	£55,950,256	£144,943,039	£233,935,822	£322,928,605	£411,921,388	£500,914,171	£589,906,954	£678,899,737	£767,892,520	£856,885,303
	3%	-£49,563,791	£39,428,993	£128,421,776	£217,414,559	£306,407,342	£395,400,125	£484,392,908	£573,385,691	£662,378,474	£751,371,257	£840,364,040
	4%	-£66,085,054	£22,907,729	£111,900,512	£200,893,295	£289,886,078	£378,878,861	£467,871,644	£556,864,427	£645,857,210	£734,849,993	£823,842,776
	5%	-£82,606,318	£6,386,465	£95,379,249	£184,372,032	£273,364,815	£362,357,598	£451,350,381	£540,343,164	£629,335,947	£718,328,730	£807,321,513
	6%	-£99,127,581	-£10,134,798	£78,857,985	£167,850,768	£256,843,551	£345,836,334	£434,829,117	£523,821,900	£612,814,683	£701,807,466	£790,800,249
	7%	-£115,648,845	-£26,656,062	£62,336,722	£151,329,505	£240,322,288	£329,315,071	£418,307,854	£507,300,637	£596,293,420	£685,286,203	£774,278,986
	8%	-£132,170,108	-£43,177,325	£45,815,458	£134,808,241	£223,801,024	£312,793,807	£401,786,590	£490,779,373	£579,772,156	£668,764,939	£757,757,722
	9%	-£148,691,372	-£59,698,589	£29,294,194	£118,286,978	£207,279,761	£296,272,544	£385,265,327	£474,258,110	£563,250,893	£652,243,676	£741,236,459
	10%	-£165,212,635	-£76,219,852	£12,772,931	£101,765,714	£190,758,497	£279,751,280	£368,744,063	£457,736,846	£546,729,629	£635,722,412	£724,715,195

Figure S.82: Incremental total cost varying the effectiveness and cost of the intervention across England over five years; low risk; 50% implementation rate; usable floor area <90m² (COPD)

		Upfront cost of intervention (per dwelling)										
		£0.00	£25.00	£50.00	£75.00	£100.00	£125.00	£150.00	£175.00	£200.00	£225.00	£250.00
Relative reduction in chronic obstructive pulmonary disease associated symptoms due to intervention	0%	£0	£6,193,476	£12,386,952	£18,580,428	£24,773,904	£30,967,380	£37,160,857	£43,354,333	£49,547,809	£55,741,285	£61,934,761
	1%	-£1,149,802	£5,043,674	£11,237,151	£17,430,627	£23,624,103	£29,817,579	£36,011,055	£42,204,531	£48,398,007	£54,591,483	£60,784,959
	2%	-£2,299,603	£3,893,873	£10,087,349	£16,280,825	£22,474,301	£28,667,777	£34,861,253	£41,054,729	£47,248,205	£53,441,681	£59,635,158
	3%	-£3,449,405	£2,744,071	£8,937,547	£15,131,023	£21,324,499	£27,517,975	£33,711,452	£39,904,928	£46,098,404	£52,291,880	£58,485,356
	4%	-£4,599,207	£1,594,269	£7,787,746	£13,981,222	£20,174,698	£26,368,174	£32,561,650	£38,755,126	£44,948,602	£51,142,078	£57,335,554
	5%	-£5,749,008	£444,468	£6,637,944	£12,831,420	£19,024,896	£25,218,372	£31,411,848	£37,605,324	£43,798,800	£49,992,276	£56,185,753
	6%	-£6,898,810	-£705,334	£5,488,142	£11,681,618	£17,875,094	£24,068,570	£30,262,047	£36,455,523	£42,648,999	£48,842,475	£55,035,951
	7%	-£8,048,612	-£1,855,136	£4,338,340	£10,531,817	£16,725,293	£22,918,769	£29,112,245	£35,305,721	£41,499,197	£47,692,673	£53,886,149
	8%	-£9,198,413	-£3,004,937	£3,188,539	£9,382,015	£15,575,491	£21,768,967	£27,962,443	£34,155,919	£40,349,395	£46,542,871	£52,736,348
	9%	-£10,348,215	-£4,154,739	£2,038,737	£8,232,213	£14,425,689	£20,619,165	£26,812,642	£33,006,118	£39,199,594	£45,393,070	£51,586,546
	10%	-£11,498,017	-£5,304,541	£888,935	£7,082,412	£13,275,888	£19,469,364	£25,662,840	£31,856,316	£38,049,792	£44,243,268	£50,436,744

Figure S.93: Incremental total cost varying the effectiveness and cost of the intervention across England over five years; low risk; 50% implementation rate; any damp problem (COPD)

Allergic rhinitis

		Upfront cost of intervention (per dwelling)										
		£0.00	£25.00	£50.00	£75.00	£100.00	£125.00	£150.00	£175.00	£200.00	£225.00	£250.00
Relative reduction in allergic rhinitis associated symptoms due to intervention	0%	£0	£3,549,465	£7,098,930	£10,648,394	£14,197,859	£17,747,324	£21,296,789	£24,846,253	£28,395,718	£31,945,183	£35,494,648
	1%	-£2,879,719	£669,746	£4,219,210	£7,768,675	£11,318,140	£14,867,605	£18,417,069	£21,966,534	£25,515,999	£29,065,464	£32,614,928
	2%	-£5,759,438	-£2,209,974	£1,339,491	£4,888,956	£8,438,421	£11,987,885	£15,537,350	£19,086,815	£22,636,280	£26,185,744	£29,735,209
	3%	-£8,639,158	-£5,089,693	-£1,540,228	£2,009,237	£5,558,701	£9,108,166	£12,657,631	£16,207,096	£19,756,560	£23,306,025	£26,855,490
	4%	-£11,518,877	-£7,969,412	-£4,419,947	-£870,483	£2,678,982	£6,228,447	£9,777,912	£13,327,376	£16,876,841	£20,426,306	£23,975,771
	5%	-£14,398,596	-£10,849,131	-£7,299,667	-£3,750,202	-£200,737	£3,348,728	£6,898,192	£10,447,657	£13,997,122	£17,546,587	£21,096,051
	6%	-£17,278,315	-£13,728,851	-£10,179,386	-£6,629,921	-£3,080,456	£469,008	£4,018,473	£7,567,938	£11,117,403	£14,666,867	£18,216,332
	7%	-£20,158,035	-£16,608,570	-£13,059,105	-£9,509,640	-£5,960,176	-£2,410,711	£1,138,754	£4,688,219	£8,237,683	£11,787,148	£15,336,613
	8%	-£23,037,754	-£19,488,289	-£15,938,824	-£12,389,360	-£8,839,895	-£5,290,430	-£1,740,965	£1,808,499	£5,357,964	£8,907,429	£12,456,894
	9%	-£25,917,473	-£22,368,008	-£18,818,544	-£15,269,079	-£11,719,614	-£8,170,149	-£4,620,685	-£1,071,220	£2,478,245	£6,027,710	£9,577,174
	10%	-£28,797,192	-£25,247,728	-£21,698,263	-£18,148,798	-£14,599,333	-£11,049,869	-£7,500,404	-£3,950,939	-£401,474	£3,147,990	£6,697,455

Figure S.104: Incremental total cost varying the effectiveness and cost of the intervention across England over five years; extreme risk; 100% implementation rate; non-decent homes (allergic rhinitis)

		Upfront cost of intervention (per dwelling)										
		£0.00	£25.00	£50.00	£75.00	£100.00	£125.00	£150.00	£175.00	£200.00	£225.00	£250.00
Relative reduction in allergic rhinitis associated symptoms due to intervention	0%	£0	£10,679,134	£21,358,268	£32,037,402	£42,716,536	£53,395,670	£64,074,804	£74,753,938	£85,433,072	£96,112,206	£106,791,340
	1%	-£8,664,097	£2,015,037	£12,694,171	£23,373,305	£34,052,439	£44,731,573	£55,410,707	£66,089,841	£76,768,975	£87,448,109	£98,127,243
	2%	-£17,328,194	-£6,649,060	£4,030,074	£14,709,208	£25,388,342	£36,067,476	£46,746,609	£57,425,743	£68,104,877	£78,784,011	£89,463,145
	3%	-£25,992,291	-£15,313,158	-£4,634,024	£6,045,110	£16,724,244	£27,403,378	£38,082,512	£48,761,646	£59,440,780	£70,119,914	£80,799,048
	4%	-£34,656,389	-£23,977,255	-£13,298,121	-£2,618,987	£8,060,147	£18,739,281	£29,418,415	£40,097,549	£50,776,683	£61,455,817	£72,134,951
	5%	-£43,320,486	-£32,641,352	-£21,962,218	-£11,283,084	-£603,950	£10,075,184	£20,754,318	£31,433,452	£42,112,586	£52,791,720	£63,470,854
	6%	-£51,984,583	-£41,305,449	-£30,626,315	-£19,947,181	-£9,268,047	£1,411,087	£12,090,221	£22,769,355	£33,448,489	£44,127,623	£54,806,757
	7%	-£60,648,680	-£49,969,546	-£39,290,412	-£28,611,278	-£17,932,144	-£7,253,010	£3,426,124	£14,105,258	£24,784,392	£35,463,526	£46,142,660
	8%	-£69,312,777	-£58,633,643	-£47,954,509	-£37,275,375	-£26,596,241	-£15,917,107	-£5,237,973	£5,441,160	£16,120,294	£26,799,428	£37,478,562
	9%	-£77,976,874	-£67,297,740	-£56,618,606	-£45,939,473	-£35,260,339	-£24,581,205	-£13,902,071	-£3,222,937	£7,456,197	£18,135,331	£28,814,465
	10%	-£86,640,972	-£75,961,838	-£65,282,704	-£54,603,570	-£43,924,436	-£33,245,302	-£22,566,168	-£11,887,034	-£1,207,900	£9,471,234	£20,150,368

Figure S.11: Incremental total cost varying the effectiveness and cost of the intervention across England over five years; extreme risk; 100% implementation rate; usable floor area <90m^2 (allergic rhinitis)

		Upfront cost of intervention (per dwelling)										
		£0.00	£25.00	£50.00	£75.00	£100.00	£125.00	£150.00	£175.00	£200.00	£225.00	£250.00
Relative reduction in allergic rhinitis associated symptoms due to intervention	0%	£0	£743,217	£1,486,434	£2,229,651	£2,972,869	£3,716,086	£4,459,303	£5,202,520	£5,945,737	£6,688,954	£7,432,171
	1%	-£602,980	£140,237	£883,454	£1,626,671	£2,369,888	£3,113,106	£3,856,323	£4,599,540	£5,342,757	£6,085,974	£6,829,191
	2%	-£1,205,960	-£462,743	£280,474	£1,023,691	£1,766,908	£2,510,125	£3,253,343	£3,996,560	£4,739,777	£5,482,994	£6,226,211
	3%	-£1,808,940	-£1,065,723	-£322,506	£420,711	£1,163,928	£1,907,145	£2,650,362	£3,393,580	£4,136,797	£4,880,014	£5,623,231
	4%	-£2,411,920	-£1,668,703	-£925,486	-£182,269	£560,948	£1,304,165	£2,047,382	£2,790,599	£3,533,817	£4,277,034	£5,020,251
	5%	-£3,014,901	-£2,271,683	-£1,528,466	-£785,249	-£42,032	£701,185	£1,444,402	£2,187,619	£2,930,836	£3,674,054	£4,417,271
	6%	-£3,617,881	-£2,874,664	-£2,131,446	-£1,388,229	-£645,012	£98,205	£841,422	£1,584,639	£2,327,856	£3,071,073	£3,814,291
	7%	-£4,220,861	-£3,477,644	-£2,734,427	-£1,991,209	-£1,247,992	-£504,775	£238,442	£981,659	£1,724,876	£2,468,093	£3,211,311
	8%	-£4,823,841	-£4,080,624	-£3,337,407	-£2,594,190	-£1,850,972	-£1,107,755	-£364,538	£378,679	£1,121,896	£1,865,113	£2,608,330
	9%	-£5,426,821	-£4,683,604	-£3,940,387	-£3,197,170	-£2,453,953	-£1,710,735	-£967,518	-£224,301	£518,916	£1,262,133	£2,005,350
	10%	-£6,029,801	-£5,286,584	-£4,543,367	-£3,800,150	-£3,056,933	-£2,313,715	-£1,570,498	-£827,281	-£84,064	£659,153	£1,402,370

Figure S.12: Incremental total cost varying the effectiveness and cost of the intervention across England over five years; extreme risk; 100% implementation rate; any damp problem (allergic rhinitis)

		Upfront cost of intervention (per dwelling)										
		£0.00	£25.00	£50.00	£75.00	£100.00	£125.00	£150.00	£175.00	£200.00	£225.00	£250.00
Relative reduction in allergic rhinitis associated symptoms due to intervention	0%	£0	£29,578,873	£59,157,746	£88,736,619	£118,315,492	£147,894,365	£177,473,238	£207,052,111	£236,630,984	£266,209,857	£295,788,730
	1%	-£7,406,685	£22,172,188	£51,751,061	£81,329,934	£110,908,807	£140,487,680	£170,066,553	£199,645,426	£229,224,299	£258,803,171	£288,382,044
	2%	-£14,813,371	£14,765,502	£44,344,375	£73,923,248	£103,502,121	£133,080,994	£162,659,867	£192,238,740	£221,817,613	£251,396,486	£280,975,359
	3%	-£22,220,056	£7,358,817	£36,937,690	£66,516,563	£96,095,436	£125,674,309	£155,253,182	£184,832,055	£214,410,928	£243,989,801	£273,568,674
	4%	-£29,626,741	-£47,868	£29,531,005	£59,109,878	£88,688,751	£118,267,624	£147,846,497	£177,425,370	£207,004,243	£236,583,116	£266,161,989
	5%	-£37,033,426	-£7,454,553	£22,124,320	£51,703,193	£81,282,065	£110,860,938	£140,439,811	£170,018,684	£199,597,557	£229,176,430	£258,755,303
	6%	-£44,440,112	-£14,861,239	£14,717,634	£44,296,507	£73,875,380	£103,454,253	£133,033,126	£162,611,999	£192,190,872	£221,769,745	£251,348,618
	7%	-£51,846,797	-£22,267,924	£7,310,949	£36,889,822	£66,468,695	£96,047,568	£125,626,441	£155,205,314	£184,784,187	£214,363,060	£243,941,933
	8%	-£59,253,482	-£29,674,609	-£95,736	£29,483,137	£59,062,010	£88,640,883	£118,219,756	£147,798,629	£177,377,502	£206,956,375	£236,535,248
	9%	-£66,660,168	-£37,081,295	-£7,502,422	£22,076,451	£51,655,324	£81,234,197	£110,813,070	£140,391,943	£169,970,816	£199,549,689	£229,128,562
	10%	-£74,066,853	-£44,487,980	-£14,909,107	£14,669,766	£44,248,639	£73,827,512	£103,406,385	£132,985,258	£162,564,131	£192,143,004	£221,721,877

Figure S.13: Incremental total cost varying the effectiveness and cost of the intervention across England over five years; low risk; 50% implementation rate; non-decent homes (allergic rhinitis)

		Upfront cost of intervention (per dwelling)										
		£0.00	£25.00	£50.00	£75.00	£100.00	£125.00	£150.00	£175.00	£200.00	£225.00	£250.00
Relative reduction in allergic rhinitis associated symptoms due to intervention	0%	£0	£88,992,783	£177,985,566	£266,978,349	£355,971,132	£444,963,915	£533,956,698	£622,949,481	£711,942,264	£800,935,047	£889,927,831
	1%	-£22,284,201	£66,708,583	£155,701,366	£244,694,149	£333,686,932	£422,679,715	£511,672,498	£600,665,281	£689,658,064	£778,650,847	£867,643,630
	2%	-£44,568,401	£44,424,382	£133,417,165	£222,409,948	£311,402,731	£400,395,514	£489,388,297	£578,381,080	£667,373,863	£756,366,646	£845,359,430
	3%	-£66,852,602	£22,140,182	£111,132,965	£200,125,748	£289,118,531	£378,111,314	£467,104,097	£556,096,880	£645,089,663	£734,082,446	£823,075,229
	4%	-£89,136,802	-£144,019	£88,848,764	£177,841,547	£266,834,330	£355,827,113	£444,819,896	£533,812,679	£622,805,462	£711,798,245	£800,791,028
	5%	-£111,421,003	-£22,428,219	£66,564,564	£155,557,347	£244,550,130	£333,542,913	£422,535,696	£511,528,479	£600,521,262	£689,514,045	£778,506,828
	6%	-£133,705,203	-£44,712,420	£44,280,363	£133,273,146	£222,265,929	£311,258,712	£400,251,495	£489,244,278	£578,237,061	£667,229,844	£756,222,627
	7%	-£155,989,404	-£66,996,621	£21,996,163	£110,988,946	£199,981,729	£288,974,512	£377,967,295	£466,960,078	£555,952,861	£644,945,644	£733,938,427
	8%	-£178,273,604	-£89,280,821	-£288,038	£88,704,745	£177,697,528	£266,690,311	£355,683,094	£444,675,877	£533,668,660	£622,661,443	£711,654,226
	9%	-£200,557,805	-£111,565,022	-£22,572,238	£66,420,545	£155,413,328	£244,406,111	£333,398,894	£422,391,677	£511,384,460	£600,377,243	£689,370,026
	10%	-£222,842,005	-£133,849,222	-£44,856,439	£44,136,344	£133,129,127	£222,121,910	£311,114,693	£400,107,476	£489,100,259	£578,093,042	£667,085,825

Figure S.14: Incremental total cost varying the effectiveness and cost of the intervention across England over five years; low risk; 50% implementation rate; usable floor area $<90m^2$ (allergic rhinitis)

		Upfront cost of intervention (per dwelling)										
		£0.00	£25.00	£50.00	£75.00	£100.00	£125.00	£150.00	£175.00	£200.00	£225.00	£250.00
**Relative reduction in allergic rhinitis associated symptoms due to intervention**	0%	£0	£6,193,476	£12,386,952	£18,580,428	£24,773,904	£30,967,380	£37,160,857	£43,354,333	£49,547,809	£55,741,285	£61,934,761
	1%	-£1,550,875	£4,642,601	£10,836,077	£17,029,553	£23,223,030	£29,416,506	£35,609,982	£41,803,458	£47,996,934	£54,190,410	£60,383,886
	2%	-£3,101,750	£3,091,727	£9,285,203	£15,478,679	£21,672,155	£27,865,631	£34,059,107	£40,252,583	£46,446,059	£52,639,535	£58,833,011
	3%	-£4,652,624	£1,540,852	£7,734,328	£13,927,804	£20,121,280	£26,314,756	£32,508,232	£38,701,708	£44,895,184	£51,088,660	£57,282,137
	4%	-£6,203,499	-£10,023	£6,183,453	£12,376,929	£18,570,405	£24,763,881	£30,957,357	£37,150,834	£43,344,310	£49,537,786	£55,731,262
	5%	-£7,754,374	-£1,560,898	£4,632,578	£10,826,054	£17,019,530	£23,213,007	£29,406,483	£35,599,959	£41,793,435	£47,986,911	£54,180,387
	6%	-£9,305,249	-£3,111,773	£3,081,703	£9,275,180	£15,468,656	£21,662,132	£27,855,608	£34,049,084	£40,242,560	£46,436,036	£52,629,512
	7%	-£10,856,123	-£4,662,647	£1,530,829	£7,724,305	£13,917,781	£20,111,257	£26,304,733	£32,498,209	£38,691,685	£44,885,161	£51,078,637
	8%	-£12,406,998	-£6,213,522	-£20,046	£6,173,430	£12,366,906	£18,560,382	£24,753,858	£30,947,334	£37,140,810	£43,334,287	£49,527,763
	9%	-£13,957,873	-£7,764,397	-£1,570,921	£4,622,555	£10,816,031	£17,009,507	£23,202,983	£29,396,460	£35,589,936	£41,783,412	£47,976,888
	10%	-£15,508,748	-£9,315,272	-£3,121,796	£3,071,680	£9,265,157	£15,458,633	£21,652,109	£27,845,585	£34,039,061	£40,232,537	£46,426,013

Figure S.155: Incremental total cost varying the effectiveness and cost of the intervention across England over five years; low risk; 50% implementation rate; any damp problems (allergic rhinitis)

GAD

		Upfront cost of intervention (per dwelling)										
		£0.00	£25.00	£50.00	£75.00	£100.00	£125.00	£150.00	£175.00	£200.00	£225.00	£250.00
**Relative reduction in generalised anxiety disorder associated symptoms due to intervention**	0%	£0	£3,549,465	£7,098,930	£10,648,394	£14,197,859	£17,747,324	£21,296,789	£24,846,253	£28,395,718	£31,945,183	£35,494,648
	1%	-£3,095,719	£453,745	£4,003,210	£7,552,675	£11,102,140	£14,651,604	£18,201,069	£21,750,534	£25,299,999	£28,849,463	£32,398,928
	2%	-£6,191,439	-£2,641,974	£907,491	£4,456,955	£8,006,420	£11,555,885	£15,105,350	£18,654,814	£22,204,279	£25,753,744	£29,303,209
	3%	-£9,287,158	-£5,737,694	-£2,188,229	£1,361,236	£4,910,701	£8,460,165	£12,009,630	£15,559,095	£19,108,560	£22,658,024	£26,207,489
	4%	-£12,382,878	-£8,833,413	-£5,283,948	-£1,734,484	£1,814,981	£5,364,446	£8,913,911	£12,463,375	£16,012,840	£19,562,305	£23,111,770
	5%	-£15,478,597	-£11,929,133	-£8,379,668	-£4,830,203	-£1,280,738	£2,268,726	£5,818,191	£9,367,656	£12,917,121	£16,466,585	£20,016,050
	6%	-£18,574,317	-£15,024,852	-£11,475,387	-£7,925,923	-£4,376,458	-£826,993	£2,722,472	£6,271,937	£9,821,401	£13,370,866	£16,920,331
	7%	-£21,670,036	-£18,120,571	-£14,571,107	-£11,021,642	-£7,472,177	-£3,922,712	-£373,248	£3,176,217	£6,725,682	£10,275,147	£13,824,611
	8%	-£24,765,756	-£21,216,291	-£17,666,826	-£14,117,361	-£10,567,897	-£7,018,432	-£3,468,967	£80,498	£3,629,962	£7,179,427	£10,728,892
	9%	-£27,861,475	-£24,312,010	-£20,762,546	-£17,213,081	-£13,663,616	-£10,114,151	-£6,564,687	-£3,015,222	£534,243	£4,083,708	£7,633,172
	10%	-£30,957,195	-£27,407,730	-£23,858,265	-£20,308,800	-£16,759,336	-£13,209,871	-£9,660,406	-£6,110,941	-£2,561,477	£987,988	£4,537,453

Figure S.16: Incremental total cost varying the effectiveness and cost of the intervention across England over five years; extreme risk; 100% implementation rate; non-decent homes (GAD)

		Upfront cost of intervention (per dwelling)										
		£0.00	£25.00	£50.00	£75.00	£100.00	£125.00	£150.00	£175.00	£200.00	£225.00	£250.00
Relative reduction in generalised anxiety disorder associated symptoms due to intervention	0%	£0	£10,679,134	£21,358,268	£32,037,402	£42,716,536	£53,395,670	£64,074,804	£74,753,938	£85,433,072	£96,112,206	£106,791,340
	1%	-£9,313,968	£1,365,165	£12,044,299	£22,723,433	£33,402,567	£44,081,701	£54,760,835	£65,439,969	£76,119,103	£86,798,237	£97,477,371
	2%	-£18,627,937	-£7,948,803	£2,730,331	£13,409,465	£24,088,599	£34,767,733	£45,446,867	£56,126,001	£66,805,135	£77,484,269	£88,163,403
	3%	-£27,941,905	-£17,262,771	-£6,583,638	£4,095,496	£14,774,630	£25,453,764	£36,132,898	£46,812,032	£57,491,166	£68,170,300	£78,849,434
	4%	-£37,255,874	-£26,576,740	-£15,897,606	-£5,218,472	£5,460,662	£16,139,796	£26,818,930	£37,498,064	£48,177,198	£58,856,332	£69,535,466
	5%	-£46,569,842	-£35,890,708	-£25,211,574	-£14,532,441	-£3,853,307	£6,825,827	£17,504,961	£28,184,095	£38,863,229	£49,542,363	£60,221,497
	6%	-£55,883,811	-£45,204,677	-£34,525,543	-£23,846,409	-£13,167,275	-£2,488,141	£8,190,993	£18,870,127	£29,549,261	£40,228,395	£50,907,529
	7%	-£65,197,779	-£54,518,645	-£43,839,511	-£33,160,378	-£22,481,244	-£11,802,110	-£1,122,976	£9,556,158	£20,235,292	£30,914,426	£41,593,560
	8%	-£74,511,748	-£63,832,614	-£53,153,480	-£42,474,346	-£31,795,212	-£21,116,078	-£10,436,944	£242,190	£10,921,324	£21,600,458	£32,279,592
	9%	-£83,825,716	-£73,146,582	-£62,467,448	-£51,788,314	-£41,109,181	-£30,430,047	-£19,750,913	-£9,071,779	£1,607,355	£12,286,489	£22,965,623
	10%	-£93,139,685	-£82,460,551	-£71,781,417	-£61,102,283	-£50,423,149	-£39,744,015	-£29,064,881	-£18,385,747	-£7,706,613	£2,972,521	£13,651,655

Figure S.17: Incremental total cost varying the effectiveness and cost of the intervention across England over five years; extreme risk; 100% implementation rate; usable floor area <90m² (GAD)

		Upfront cost of intervention (per dwelling)										
		£0.00	£25.00	£50.00	£75.00	£100.00	£125.00	£150.00	£175.00	£200.00	£225.00	£250.00
Relative reduction in generalised anxiety disorder associated symptoms due to intervention	0%	£0	£743,217	£1,486,434	£2,229,651	£2,972,869	£3,716,086	£4,459,303	£5,202,520	£5,945,737	£6,688,954	£7,432,171
	1%	-£648,208	£95,009	£838,226	£1,581,443	£2,324,660	£3,067,878	£3,811,095	£4,554,312	£5,297,529	£6,040,746	£6,783,963
	2%	-£1,296,416	-£553,199	£190,018	£933,235	£1,676,452	£2,419,669	£3,162,887	£3,906,104	£4,649,321	£5,392,538	£6,135,755
	3%	-£1,944,624	-£1,201,407	-£458,190	£285,027	£1,028,244	£1,771,461	£2,514,679	£3,257,896	£4,001,113	£4,744,330	£5,487,547
	4%	-£2,592,832	-£1,849,615	-£1,106,398	-£363,181	£380,036	£1,123,253	£1,866,470	£2,609,688	£3,352,905	£4,096,122	£4,839,339
	5%	-£3,241,040	-£2,497,823	-£1,754,606	-£1,011,389	-£268,172	£475,045	£1,218,262	£1,961,480	£2,704,697	£3,447,914	£4,191,131
	6%	-£3,889,248	-£3,146,031	-£2,402,814	-£1,659,597	-£916,380	-£173,163	£570,054	£1,313,271	£2,056,489	£2,799,706	£3,542,923
	7%	-£4,537,457	-£3,794,239	-£3,051,022	-£2,307,805	-£1,564,588	-£821,371	-£78,154	£665,063	£1,408,280	£2,151,498	£2,894,715
	8%	-£5,185,665	-£4,442,448	-£3,699,230	-£2,956,013	-£2,212,796	-£1,469,579	-£726,362	£16,855	£760,072	£1,503,290	£2,246,507
	9%	-£5,833,873	-£5,090,656	-£4,347,438	-£3,604,221	-£2,861,004	-£2,117,787	-£1,374,570	-£631,353	£111,864	£855,081	£1,598,299
	10%	-£6,482,081	-£5,738,864	-£4,995,647	-£4,252,429	-£3,509,212	-£2,765,995	-£2,022,778	-£1,279,561	-£536,344	£206,873	£950,090

Figure S.18: Incremental total cost varying the effectiveness and cost of the intervention across England over five years; extreme risk; 100% implementation rate; any damp problem (GAD)

Relative reduction in generalised anxiety disorder associated symptoms due to intervention	Upfront cost of intervention (per dwelling)										
	£0.00	£25.00	£50.00	£75.00	£100.00	£125.00	£150.00	£175.00	£200.00	£225.00	£250.00
0%	£0	£29,578,873	£59,157,746	£88,736,619	£118,315,492	£147,894,365	£177,473,238	£207,052,111	£236,630,984	£266,209,857	£295,788,730
1%	-£7,962,241	£21,616,632	£51,195,505	£80,774,378	£110,353,250	£139,932,123	£169,510,996	£199,089,869	£228,668,742	£258,247,615	£287,826,488
2%	-£15,924,483	£13,654,390	£43,233,263	£72,812,136	£102,391,009	£131,969,882	£161,548,755	£191,127,628	£220,706,501	£250,285,374	£279,864,247
3%	-£23,886,724	£5,692,149	£35,271,022	£64,849,895	£94,428,768	£124,007,641	£153,586,514	£183,165,387	£212,744,260	£242,323,133	£271,902,005
4%	-£31,848,966	-£2,270,093	£27,308,780	£56,887,653	£86,466,526	£116,045,399	£145,624,272	£175,203,145	£204,782,018	£234,360,891	£263,939,764
5%	-£39,811,207	-£10,232,334	£19,346,539	£48,925,412	£78,504,285	£108,083,158	£137,662,031	£167,240,904	£196,819,777	£226,398,650	£255,977,523
6%	-£47,773,449	-£18,194,576	£11,384,297	£40,963,170	£70,542,043	£100,120,916	£129,699,789	£159,278,662	£188,857,535	£218,436,408	£248,015,281
7%	-£55,735,690	-£26,156,817	£3,422,056	£33,000,929	£62,579,802	£92,158,675	£121,737,548	£151,316,421	£180,895,294	£210,474,167	£240,053,040
8%	-£63,697,931	-£34,119,058	-£4,540,185	£25,038,688	£54,617,561	£84,196,433	£113,775,306	£143,354,179	£172,933,052	£202,511,925	£232,090,798
9%	-£71,660,173	-£42,081,300	-£12,502,427	£17,076,446	£46,655,319	£76,234,192	£105,813,065	£135,391,938	£164,970,811	£194,549,684	£224,128,557
10%	-£79,622,414	-£50,043,541	-£20,464,668	£9,114,205	£38,693,078	£68,271,951	£97,850,824	£127,429,697	£157,008,570	£186,587,443	£216,166,316

Figure S.19: Incremental total cost varying the effectiveness and cost of the intervention across England over five years; low risk; 50% implementation rate; non-decent homes (GAD)

Relative reduction in generalised anxiety disorder associated symptoms due to intervention	Upfront cost of intervention (per dwelling)										
	£0.00	£25.00	£50.00	£75.00	£100.00	£125.00	£150.00	£175.00	£200.00	£225.00	£250.00
0%	£0	£88,992,783	£177,985,566	£266,978,349	£355,971,132	£444,963,915	£533,956,698	£622,949,481	£711,942,264	£800,935,047	£889,927,831
1%	-£23,955,680	£65,037,103	£154,029,886	£243,022,669	£332,015,452	£421,008,235	£510,001,018	£598,993,801	£687,986,584	£776,979,367	£865,972,150
2%	-£47,911,361	£41,081,423	£130,074,206	£219,066,989	£308,059,772	£397,052,555	£486,045,338	£575,038,121	£664,030,904	£753,023,687	£842,016,470
3%	-£71,867,041	£17,125,742	£106,118,525	£195,111,308	£284,104,091	£373,096,874	£462,089,658	£551,082,441	£640,075,224	£729,068,007	£818,060,790
4%	-£95,822,721	-£6,829,938	£82,162,845	£171,155,628	£260,148,411	£349,141,194	£438,133,977	£527,126,760	£616,119,543	£705,112,326	£794,105,109
5%	-£119,778,401	-£30,785,618	£58,207,165	£147,199,948	£236,192,731	£325,185,514	£414,178,297	£503,171,080	£592,163,863	£681,156,646	£770,149,429
6%	-£143,734,082	-£54,741,299	£34,251,485	£123,244,268	£212,237,051	£301,229,834	£390,222,617	£479,215,400	£568,208,183	£657,200,966	£746,193,749
7%	-£167,689,762	-£78,696,979	£10,295,804	£99,288,587	£188,281,370	£277,274,153	£366,266,936	£455,259,720	£544,252,503	£633,245,286	£722,238,069
8%	-£191,645,442	-£102,652,659	-£13,659,876	£75,332,907	£164,325,690	£253,318,473	£342,311,256	£431,304,039	£520,296,822	£609,289,605	£698,282,388
9%	-£215,601,122	-£126,608,339	-£37,615,556	£51,377,227	£140,370,010	£229,362,793	£318,355,576	£407,348,359	£496,341,142	£585,333,925	£674,326,708
10%	-£239,556,803	-£150,564,020	-£61,571,237	£27,421,547	£116,414,330	£205,407,113	£294,399,896	£383,392,679	£472,385,462	£561,378,245	£650,371,028

Figure S.20: Incremental total cost varying the effectiveness and cost of the intervention across England over five years; low risk; 50% implementation rate; usable floor area <90m^2 (GAD)

		Upfront cost of intervention (per dwelling)										
		£0.00	£25.00	£50.00	£75.00	£100.00	£125.00	£150.00	£175.00	£200.00	£225.00	£250.00
Relative reduction in generalised anxiety disorder associated symptoms due to intervention	0%	£0	£6,193,476	£12,386,952	£18,580,428	£24,773,904	£30,967,380	£37,160,857	£43,354,333	£49,547,809	£55,741,285	£61,934,761
	1%	-£1,667,202	£4,526,274	£10,719,750	£16,913,226	£23,106,703	£29,300,179	£35,493,655	£41,687,131	£47,880,607	£54,074,083	£60,267,559
	2%	-£3,334,404	£2,859,072	£9,052,548	£15,246,025	£21,439,501	£27,632,977	£33,826,453	£40,019,929	£46,213,405	£52,406,881	£58,600,357
	3%	-£5,001,606	£1,191,871	£7,385,347	£13,578,823	£19,772,299	£25,965,775	£32,159,251	£38,352,727	£44,546,203	£50,739,679	£56,933,155
	4%	-£6,668,807	-£475,331	£5,718,145	£11,911,621	£18,105,097	£24,298,573	£30,492,049	£36,685,525	£42,879,001	£49,072,477	£55,265,953
	5%	-£8,336,009	-£2,142,533	£4,050,943	£10,244,419	£16,437,895	£22,631,371	£28,824,847	£35,018,323	£41,211,799	£47,405,276	£53,598,752
	6%	-£10,003,211	-£3,809,735	£2,383,741	£8,577,217	£14,770,693	£20,964,169	£27,157,645	£33,351,122	£39,544,598	£45,738,074	£51,931,550
	7%	-£11,670,413	-£5,476,937	£716,539	£6,910,015	£13,103,491	£19,296,967	£25,490,444	£31,683,920	£37,877,396	£44,070,872	£50,264,348
	8%	-£13,337,615	-£7,144,139	-£950,663	£5,242,813	£11,436,290	£17,629,766	£23,823,242	£30,016,718	£36,210,194	£42,403,670	£48,597,146
	9%	-£15,004,817	-£8,811,341	-£2,617,865	£3,575,612	£9,769,088	£15,962,564	£22,156,040	£28,349,516	£34,542,992	£40,736,468	£46,929,944
	10%	-£16,672,019	-£10,478,542	-£4,285,066	£1,908,410	£8,101,886	£14,295,362	£20,488,838	£26,682,314	£32,875,790	£39,069,266	£45,262,742

Figure S.216: Incremental total cost varying the effectiveness and cost of the intervention across England over five years; low risk; 50% implementation rate; any damp problem (GAD)
